# Study on the damage resistance and deterioration behavior of GO concrete under the harsh environment

**DOI:** 10.1371/journal.pone.0284186

**Published:** 2023-04-12

**Authors:** Chengqin Chen, Wei Zhang, Hongjuan Wu, Chenggui Chen, Bin Ma

**Affiliations:** 1 School of Civil Engineering, Northwest Minzu University, Lanzhou, China; 2 Key Laboratory of New Building Materials and Building Energy of Gansu Province, Lanzhou, China; Beijing University of Technology, CHINA

## Abstract

To exploretheeffects of physical, mechanical, anti-deterioration properties of graphene oxide (GO) on cement-based cementitious materials, GO sheet dispersions areprepared by the improved Hummers method and ultrasonic dispersion method. The influence of theGO content on the compressive and flexural strengths of cement paste is investigated, and the penetration process of chloride ions in graphene oxide concrete is discussed by the electric accelerated erosion method. Combined with the rapid freeze-thaw test, the deterioration of graphene oxide concrete ismethodically analyzed. Theobtained results reveal that an appropriate amount of GO improves both the compressive and flexural strengths of cement pastev. In the chloride environment, the chloride diffusion coefficient of 0.03% GO concrete is 18.75% less than that of ordinary concrete.Under the action of freeze-thaw cycles, with the increase of salt freezing times, the deterioration mode of GO concrete is a combination of mortar shedding, micro-crack expansion, denudation, and block shedding; The stress-strain curve of the specimen tends to be flat with the growth of salt freezing times. The peak stress gradually lessens, the peak strain gradually grows, and the elastic modulus remarkably reduces. Compared with ordinary cement paste, theGO is capable of promoting the growth of cement paste hydration crystals, changing the size and shape of crystals, and realizingthe regulation of cement paste microstructure. Incorporating an appropriate amount of theGO could promote the cement hydration process and enhance the chemical water-binding amount in the cement paste. The optimal GO content is reported to be 0.03% of the cement mass.

## 1. Introduction

Concrete is extensively utilized worldwide due to its abundant source of materials, low price, and simple construction. With the long-term development of domestic infrastructure construction, the durability of concrete structures has become a hot issue in civil engineering. The structure’s durability is directly related to the project’s safety and even the national economy’s social benefits, so it paid more and more attention [1, 2]. Mehta [3] pointed out the causes of substantial damage by the significance of steel corrosion, freeze-thaw damage, and physical changes in the erosion environment. Therefore, the freeze-thaw damage to concrete is an essential factor affecting durability in the severe cold region of Northeast China, and it is also one of the main factors causing damage in engineering structures.

In the Northeast China, buildings are often subjected to freezing disaster because of the low temperature in winter temperature, and when the temperature rises, the freezing and concreting buildings begin to melt, and the buildings are subjected to repeated freezing and thawing cycles, make making buildings more vulnerable to damage [1–3]. Because of the concrete structure in the environment below the freezing point, the water in the inner pores will freeze, causing the volume expansion, causing the migration of the unfrozen water, and the water will exert pressure on the concrete, the reaction of concrete to and water is causes a tensile force. The concrete itself is a brittle material, when this kind of force reaches a certain degree, the destruction of concrete occurs the destruction [4, 5] as this kind of force reaches a certain degree. Moreover Additionally, the frost resistance of concrete is still a very important crucial aspect of concrete durability. At home and abroad, The frost resistance of concrete is often used employed as the main basic standard or comprehensive index to evaluate the durability of concrete at home and abroad. By this view, this paper aims to analyzes the effect of GO on improving the frost resistance of concrete by freezing-thawing cycle experiment.

In recent decades, nanomaterial science has promptly developed [6–8]. Many scholars at home and abroad have applied nanomaterials(such as nano-SiO_2_, nano-CaCO_3_, andnano-TiO_2_) to cement concrete and examined their influences on the concrete strength, microstructure, cement hydration process, and whether it is beneficial to improve the durability of concrete. With an in-depth realizing the basic theory of cement-based materials at the nanoscale (such as the structure and mechanical properties of the primary hydration phases, the source of cement cohesion, cement hydration, and concrete interface), nanoscience has made noticeable progress in cement-based materials. On the one hand, nanomaterials as fillers could effectively fill the pores of the concrete, reduce the number of harmful spores in the cement matrix, and enhance the compactness of the cement matrix. On the other hand, they can react with Ca(OH)_2_ with low strength in hydration products to produce more hydration calcium silicate gel (C-S-H) and ettringite (AFt), thereby enhancing the strength of cement-based materials and improving their durability. Additionally, nanomaterials have a series of unique properties such as electricity, heat, electromagnetic, and light. The addition of nanomaterials to cement-based materials providesad-based composites have some unique functions, which brings a new approach to realizing multi-functional and high-performance concrete production.

Graphene is the thinnest two-dimensional nanomaterial with excellent mechanical, electrical, and thermal properties [9–15]. The conducted investigations have shown that graphene improves the mechanical strength, toughness, and durability of cement-based materials. Simultaneously, its excellent electrical and thermal conductivity properties provide cement-based materials with specific functional properties [16–21]. The GO is a derivative of graphene. Compared with graphene, there are many oxygen-containing functional groups on the surface of GO (such as hydroxyl, carboxyl, and epoxy group). The presence of these hydrophilic oxygen-containing functional groups makes it have better dispersion ability in an aqueous solution. However, these functional groups also slightly lessen their mechanical properties, and the deterioration of the conjugated bond between carbon atoms in the preparation process leads to structural defects, as well as damaging its unique thermal and electrical conductivities [16]. Therefore, it is necessary to choose between them according to the required performance in practical applications. The successful preparation of graphene and its derivatives offers a potentially new candidate for nano-additives exploited in cement-based materials. Xu et al. [17] and Francesco et al. [18] prepared nanoscale GO dispersions via improved hummers and ultrasonic dispersion methods. The gained results indicated that incorporating GO alter change the microstructure of crystals in cement stone, promote the formation of neat and regular nanoscale micro crystals of cement hydration products, and achieve the effect of strengthening and toughening. Lin et al. [22], Mohammed et al. [23], and Shang et al. [24] prepared graphene dispersion suspension by nitric acid oxidation and ultrasonic method and explored the effect of graphene content on the mechanical properties and microstructure of the cement paste. Pan et al. [25] prepared the GO by Hummers and ultrasonic dispersion methods and the corrosion resistance of GO-based cement mortar subjected to different erosion environments. Lu et al. [26] assessed the influence of the GO on the hydration and mechanical properties of fly ash cement by XRD, hydration heat, and SEM. Sheng et al. [27] investigated the compressive strength, pore structure, and microstructure of GO on foamed cement materials by preparing GO/foamed cement-based materials. Although the research on GO cement-based materials has made some progress, the mechanical properties of GO concrete specimens under the combined action of chloride and freeze-thaw are less examined.

In recent years, many experts and investigators in China and abroad have not only limited the mselves to enhancing the mechanical properties of cement-based materials in the research and application of graphene and its derivatives mixed with cement-based composites, but also carried out an in-depth exploration of its durability, conductive and thermal conductivity, pressure sensitivity, electromagnetic shielding, and other aspects [28–31]. However, as a relatively new research direction, many problems can be solved in theoretical research and application. Therefore, in this paper, the improved Hummers method and ultrasonic dispersion method are employed to prepare GO dispersion, the effect of various dosages of GO on the hydration and mechanical properties of cement paste is investigated, and the mechanism of GO dispersion regulating hydration products is discussed, and the effect of GO on hydration products examined. The influence of concrete working performance, chloride ion corrosion resistance, and freezing resistance performance, the corrosion behavior and damage process of chloride ions in concrete under the action of chloride ion erosion, as well as freezing and thawing of concrete specimens with different GO contents are carried out, and the chlorine ion in concrete is analyzed. The depth of theion erosion and the content of chloride ions, the degradation law of compressive strength, and the deformation characteristics of GO concrete under the action of freeze-thaw are discussed, and the specific application and research of GO in concrete durability are provided.

## 2. Experimental research

### 2.1.Experimental material

The used graphite in the test (particle size <10um) has been produced by Nanjing Xianfeng Nanomaterial Technology Co., Ltd. Sodium nitrate (NaNO_3_), concentrated sulfuric acid (98%), potassium permanganate (KMnO_4_), phosphoric acid (H_3_PO_4_), hydrogen peroxide (H_2_O_2_) and hydrochloric acid (HCl) are all analytically pure, which are provided by Dalian Kexin Chemical Co., Ltd. The DK-PC type polycarboxylate superplasticizer is produced by Dalian Jianke North Chemical Co., Ltd. It is a brownish-yellow liquid with a water reduction rate of 36%, a solids content of 35%, and a content of 1.2% of the cement mass fraction. The test cement is Xiao ye tian PO·42.5 Ordinary Portland cement, and its chemical composition is detailed in Table 1.

**Table 1:** Chemical composition of cement.

Chemical components	CaO	SiO_2_	Al_2_O_3_	Fe_2_O_3_	MgO	SO_3_
Content /%	59.30	21.91	6.27	3.78	1.64	2.41

### 2.2. Preparation of GO dispersion

Graphite is oxidized by the improved Hummers method [32, 33]. 5g Graphite powder, 3g NaNO_3_ powder, 125 mL concentrated sulfuric acid, and 15 mL phosphoric acid was stirred evenly in the ice bath, and then 20 g KMnO_4_ was slowly added. After continuous stirring for 3hours, the color of the solution became dark green. The solution was continuously stirred in a water bath at 40°C for 2h, and then the solution became a viscous brown liquid. Then 230mL distilled water was added to the solution, the temperature rose to about 98°C, stirring for 0.5hours, cooling to room temperature, and then 50mL H_2_O_2_ was added to the solution to become bright yellow. The product was centrifuged and washed with 10% HCl and distilled water until the solution pH was close to 7. The obtained GO was dissolved in distilled water and treated with 300W ultrasonic cleaner for 60 mins to obtain GO dispersion. The mass concentration was set to 5mg / mL. The technical dispersion indexes have been provided in Table 2.

**Table 2:** Dispersion technical index of GO.

pH	Thickness /nm	Monolayer rate /%	Oxygen content /%	Sulfur content /%
6.8	0.05–1.0	≥99	≤49	≤3

### 2.3. Specimen preparation

An SX2-8-10 chamber electric furnace was utilized for high-temperature calcination. Before this, the sampled powder was sealed into a crucible and placed in the furnace for calcination. The crucible was fetched out for cooling and further processing only after the temperature had risen to the specified temperature and sustained for 2hours.

According to the existing research results [34, 35], the preparation of concrete specimens considers four kinds of GO content: 0.00%, 0.01%, 0.03%,and 0.05%, denoted asGO-0, GO-1, GO-3, and GO-5.The standard sample size is *100 mm×100 mm×100 mm*, and each group consists of 3 specimens, a total of 45. The mixture ratio of the specimen was designed as: *m*_*(cement)*_: *m*_*(water)*_: *m*_*(sand)*_: *m*_*(stone)*_: *m*_*(water reducing agent)*_ = 420: 168: 782: 1080: 2.1. After molding, the specimen was moulded for 24 h and placed in a standard curing box for 28days for the test.

### 2.4. Experimental method

#### 2.4.1. Chloride ion permeability test

In order to investigate the distribution of chloride ion content in the GO-modified concrete, this section explores the law of chloride ion erosion in GO concrete by the method of constant voltage electrochemical accelerated corrosion. For this purpose, three levels of the acceleration voltage (0, 20, and 30V) are set up, and the specific test groups are presented in Table 3.

**Table 3:** Experimental conditions.

Test Method	Voltage /V	Erosion Time /h	Test Index
Electrically accelerated erosion	20	12	Penetration depth Content
	30	24	
		36	
Natural immersion	0	36	

#### 2.4.2. Anti-freezing test

**Fig 1:**
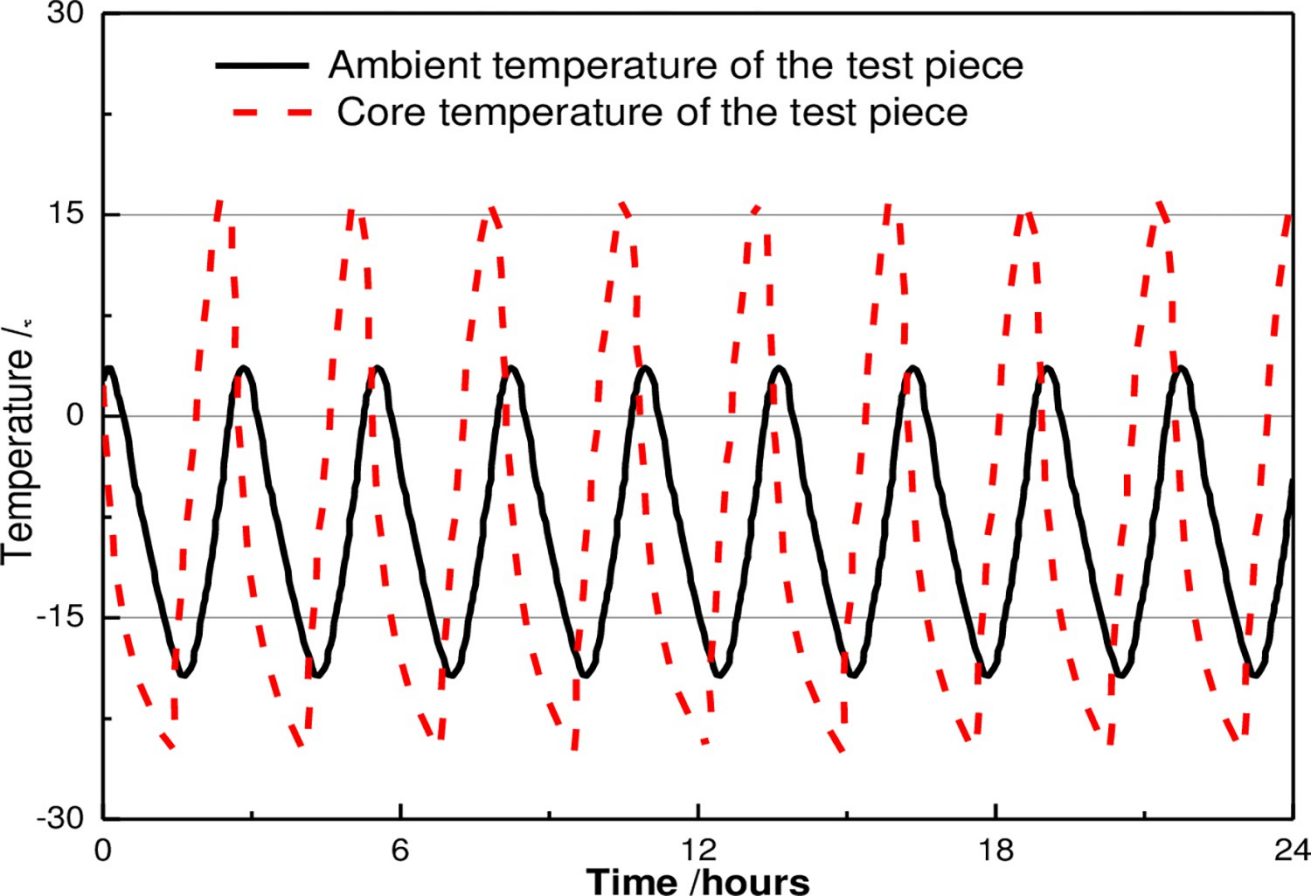
Temperature change curve during the freeze-thaw cycles.

Under the relevant provisions of the test method for long-term performance and durability of ordinary concrete(GBJ82-85), the freeze-thaw cycle test was on ducted by the TDR-16 rapid freeze-thaw testing machine, and the dynamic modulus of concrete under the freeze-thaw cycle was measured by DT-12 dynamic modulus tester. The schematic representations of the freeze-thaw cycle device and the temperature change curve are demonstrated in Fig 1.

Each freeze-thaw cycle of the freeze-thaw cycle test shall be completed within 2-4h, and the melting time shall not be less than 1/4 of the whole freeze-thaw cycle time. At the end of freezing and melting, the center temperature of the test piece shall be controlled at—17 ± 2°C and 8 ± 2°C, respectively. The time taken for each test piece to drop from 6°C to—15°Cshould not be less than 1/2 of the freezing time, while the required time to increase from—15°C to 6°Cshould not be less than 1/2 of the melting time, and the discrepancy between the inside and outside temperatures of the test piece should not exceed 28°C. The conversion time of freezing and melting should not exceed 10min.

#### 2.4.3. Mechanical property measurement

After putting the concrete specimen into a freeze-thaw test box, take out one group every 25 cycles. The YAW-YAW2000A type 200t microcomputer-controlled electro-hydraulic servo pressure testing machine is utilized to test the flexural strength and compressive strength of concrete under the current standard condition for Highway Engineering Cement and Cement Concrete Test Procedure(JTGE30-2005). The loading speed is controlled at 2400 ± 200N / s.

#### 2.4.4. Microstructure analysis

The morphology and microstructure of some selected samples of GO cement composites are examined via the scanning electron microscope(SEM) technique. The specimen was cured to the test age and broken. The block of *1cm × 1cm × 1cm* was put into anhydrous ethanol to terminate hydration for 48 hours, and the test section of the specimen was not treated. The surface fracture was covered with a thin layer of gold, and SEM micrographs were obtained with a SUPRA 55 SAPPHIRE.

#### 2.4.5. XRD analysis

In the current investigation, the mineral composition of samples at various ages was determined by an X-ray diffractometer(XRD). Before the test, the sample was made into a cube with a side length of about 1 cm. In the diffraction test, the test radius was greater than200 mm, the scanning speed was 10° /min, and the scanning angle (2θ) was set as10–90°.

#### 2.4.6. Differential scanning calorimetry

In this paper, the content of hydration products (hydrated calcium silicate gel (C-S-H) and calcium hydroxide (CH)) in GO-modified cement was determined by TG-DSC STA 409 thermal gravimetric analyzer. First, samples are taken from the core part of the specimen, and the hydration was terminated by acetone for one day. The prepared samples were then dried at 105°C for 4 h in a vacuum drying oven and ground to a fineness of fewer than 150 μm. During the TG analysis, the temperature rate was set equal to15°C /min, the heating atmosphere was nitrogen, and the gas flow rate was set as 50 mL/min.

The reference weight calculated by the percentage of CH in the sample is the mass of the sample after burning at 950° C for 30 minutes. TheCH content is could be also calculated by the formula Eqs (1) and (2):

$$CH=\frac{\Delta m}{m_{0}}\times \frac{M_{CH}}{M}\times 100\%$$ (1)

$$Ca{\left(OH\right)}_{2}\to CaO+H_{2}O↑$$ (2)

Where *Δm* is the mass loss in the temperature range of 420–490°C; *m*_*0*_ is the reference weight, which is the mass of the sample after burning at 950°C for 30 min. *M*_*CH*_ and *M* are the molar mass of Ca(OH)_2_ and H_2_O, respectively.

All the experimental samples are shown in Table 4.

**Table 4:** Experimental conditions.

Test	Specimen size	Test conditions	Test Index
Chloride ion permeability test	Φ10 × 5 cm	Acceleration voltages: 0V, 20V, and 30V	Penetration depth, Content
Antifreezing test	10 cm×10 cm×40 cm	Center temperature: - 17 ± 2°C and 8 ± 2°C Temperature range: 6°C to—15°C	Mass loss Mechanical property, Stress-strain curve
Mechanical property measurement	10 cm×10 cm×40 cm	Loading speed: 2400 ± 200N / s	Flexural strength, Compressive strength
Microstructure analysis	1cm × 1cm × 1cm	Terminate hydration:48 hours	C-S-H gel, CH crystals, Micro-pores and cracks
XRD analysis	1cm × 1cm × 1cm	Test radius: ≥ 200 mm, Scanning speed: 10° / min, Scanning angle 2θ: 10–90°.	CH crystal, Aft crystal, AFM crystal, C2S, C3S, C3A, and C4AF
Differential Scanning Calorimetry	Fewer than 150 μm	Dried at 105°C for 4 h, Temperature programmed rate was 15°C / min, Gas flow rate:50 mL/min	Weight loss

## 3. Test results and discussion

### 3.1. Physical and mechanical properties

#### 3.1.1. Flowability

**Fig 2:**
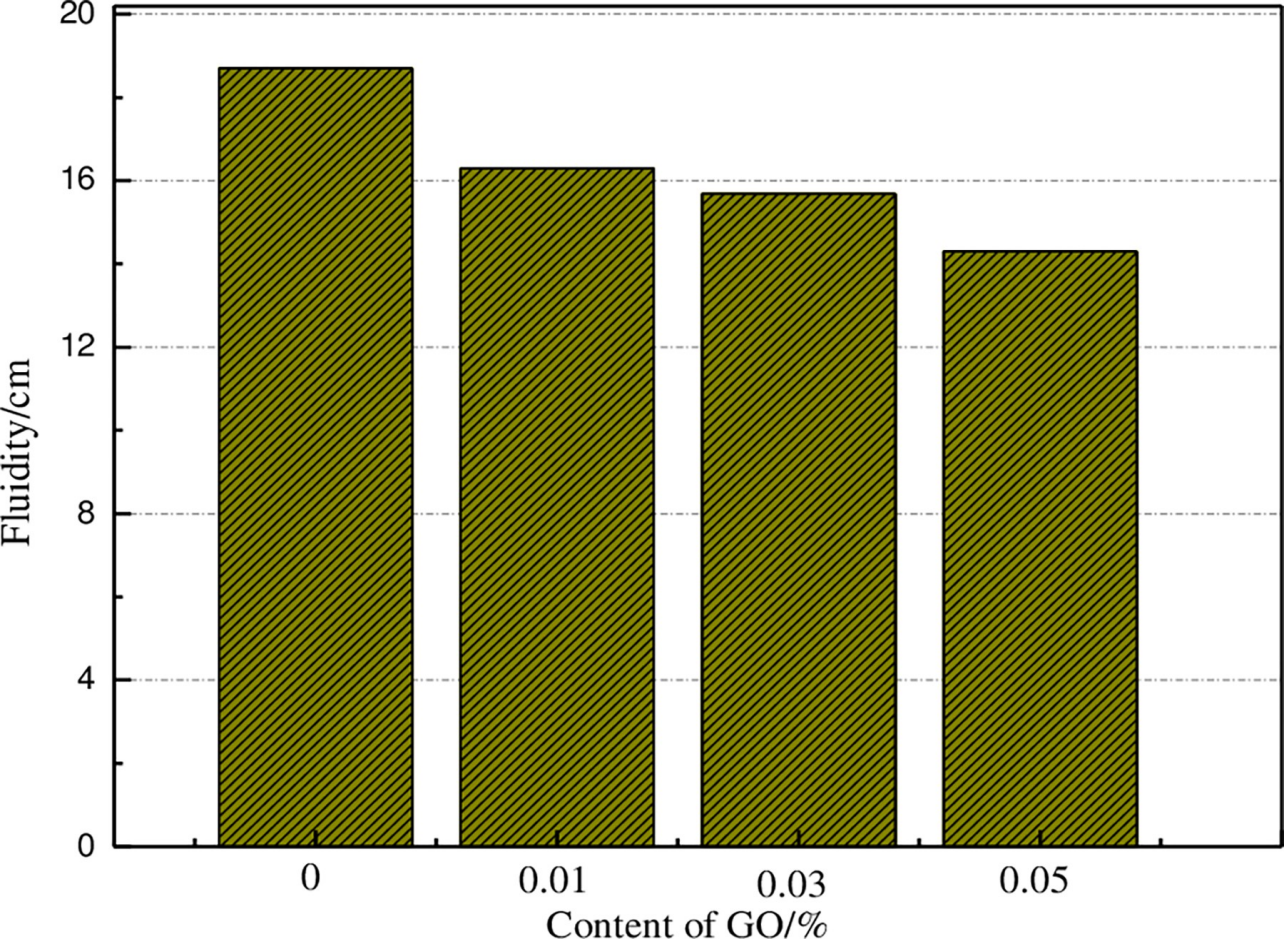
The fluidity of cement slurry under different GO content.

As illustrated in Fig 2, the fluidity of cement mortar reduces with the growth of the GO content. The reason for this fact is that the GO presents a multi-layer two-dimensional honeycomb network structure with a large specific surface area. The outer surface and edge contain hydroxyl, carboxyl, epoxy, and other groups. Many oxygen-containing groups will make GO hydrophilic and combine with H atoms in water. With the growth of the GO content, more water is consumed, thereby reducing the fluidity of the sample. Additionally, the nano-size effect of GO affects the adsorption double electron layer formed by cement particles and water reducer, which leads to the reduction of repulsion between cement particles and the growth of the fluidity. The obtained results from this study are consistent with previous investigations [36].

#### 3.1.2. Mechanical property

**Fig 3:**
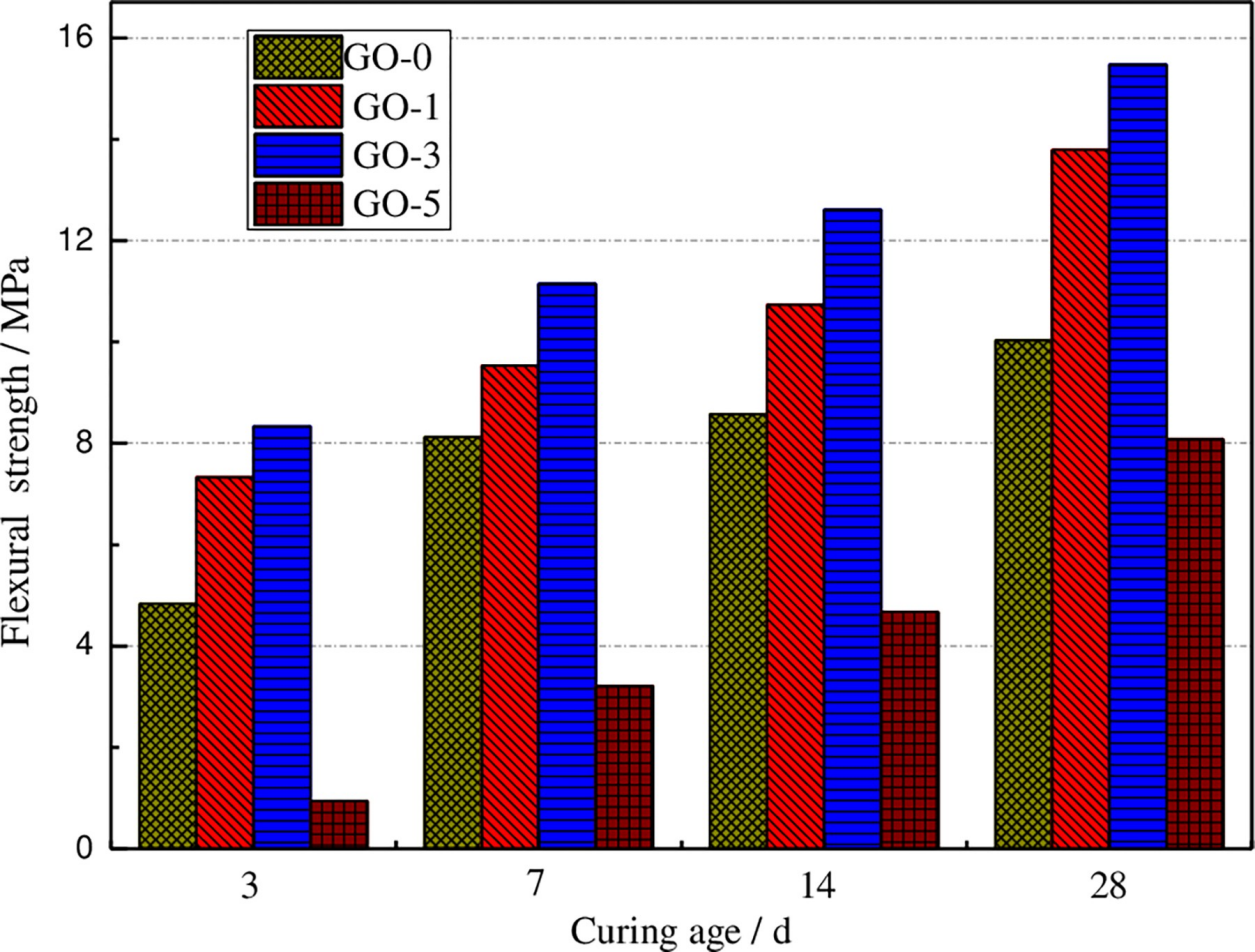
Flexural strength of GO cement paste under various curing ages.

The flexural strength values of different GO cement paste specimens at various curing ages have been illustrated in Fig 3.

It can be seen from Fig 3 that after adding a small amount of the GO, with the increase of age, the mechanical strength of cement paste becomes higher than that of ordinary specimens, particularly when the contents of 0.01% and 0.03% concrete are added. The flexural strength of the GO cement paste rises with the growth of the curing age. The plotted results in Fig 6 reveal that with the increase of the GO content, the flexural strength increases first and then decreases. When the GO content is about 0.03%, the flexural strength of the specimen reaches the maximum. Compared with ordinary concrete specimens, the growth of 3, 7, 14, and28 days in order are 17.38%, 12.73%, 16.35%, and 18.05%. The flexural strength lessens as the GO content grows compared with ordinary specimens. It indicates that for high GO content, due to the enrichment of the C element, it cannot be well dispersed into cement-based materials, resulting in the reduction ofd mechanical properties.

**Fig 4:**
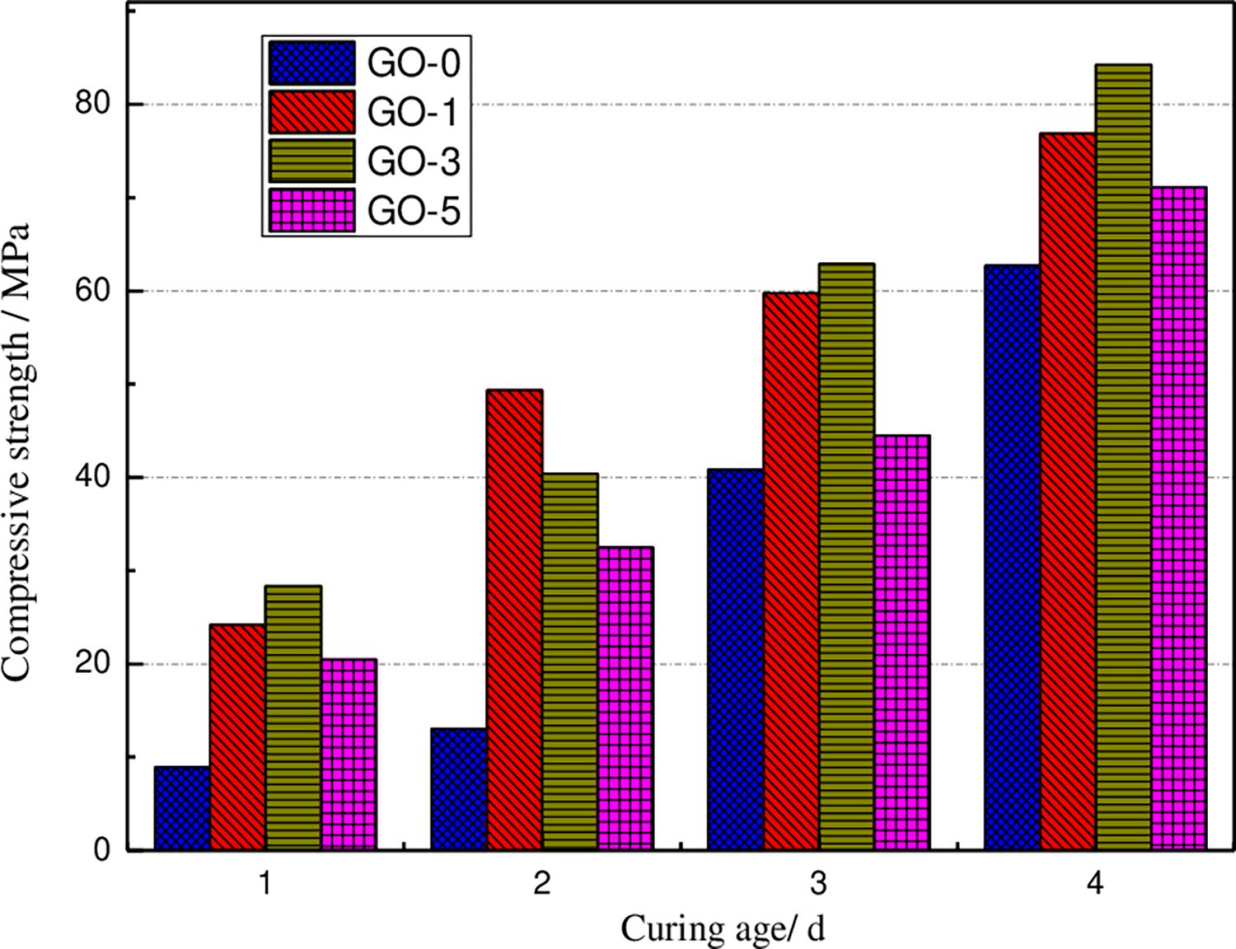
Compressive strength of GO cement paste under various curing ages.

The compressive strength values of GO cement paste under for various curing ages are illustrated in Fig 4. The demonstrated plots reveal that for the case of the GO content is equal to0.03%,the compressive strength of the specimen reaches the maximum. Compared with the ordinary concrete specimen, the growth of compressive strength of GO concrete for curing times 3,7,14, and 28 d are reported as 14.74%, 22.71%, 6.8% and 12.31%, respectively. Compared with the upper flexural strength, the amount of growth is small, indicating that the incorporation of 0.03% GO improves the toughness of cement paste. It can be seen from Table 4 that the compressive strength of GO cement pastes with 0.01%, 0.03%, and 0.05% GO enhances by 12.51%, 14.74%, and 9.13%, respectively, compared with that of ordinary cement pastes at the curing age of 3 d. The flexural strength of the GO cement pastes with 0.01% and 0.03% GO in order increases by 10.84% and 17.38% respectively, while the flexural strength of 0.05%GO reduces by 17.38%. The compressive strength of cement paste mixed with 0.01%, 0.03%, and 0.05% GO increases by 29.09%, 22.71%, and 16.09%, respectively compared with ordinary cement paste after seven days of curing. The flexural strength of cement paste mixed with 0.01% and 0.03% GO increased by 5.94% and 12.73%, respectively, while the flexural strength of 0.05% GO decreased by 18.68%. At the curing age of 28 d, the compressive strength of GO cement paste with 0.01%, 0.03%, and 0.05% is7.8%, 12.31%, and 5.56% higher than ordinary cement paste, respectively. It can be seen that adding a small amount of GO can improve the compressive strength of cement paste at an early age. Therefore, using GO as an early strength reinforcement of cement provides a new direction for improving the early performance of cement-based composites.

### 3.2. Chloride ion permeability

#### 3.2.1. Chloride penetration depth of concrete

**Fig 5:**
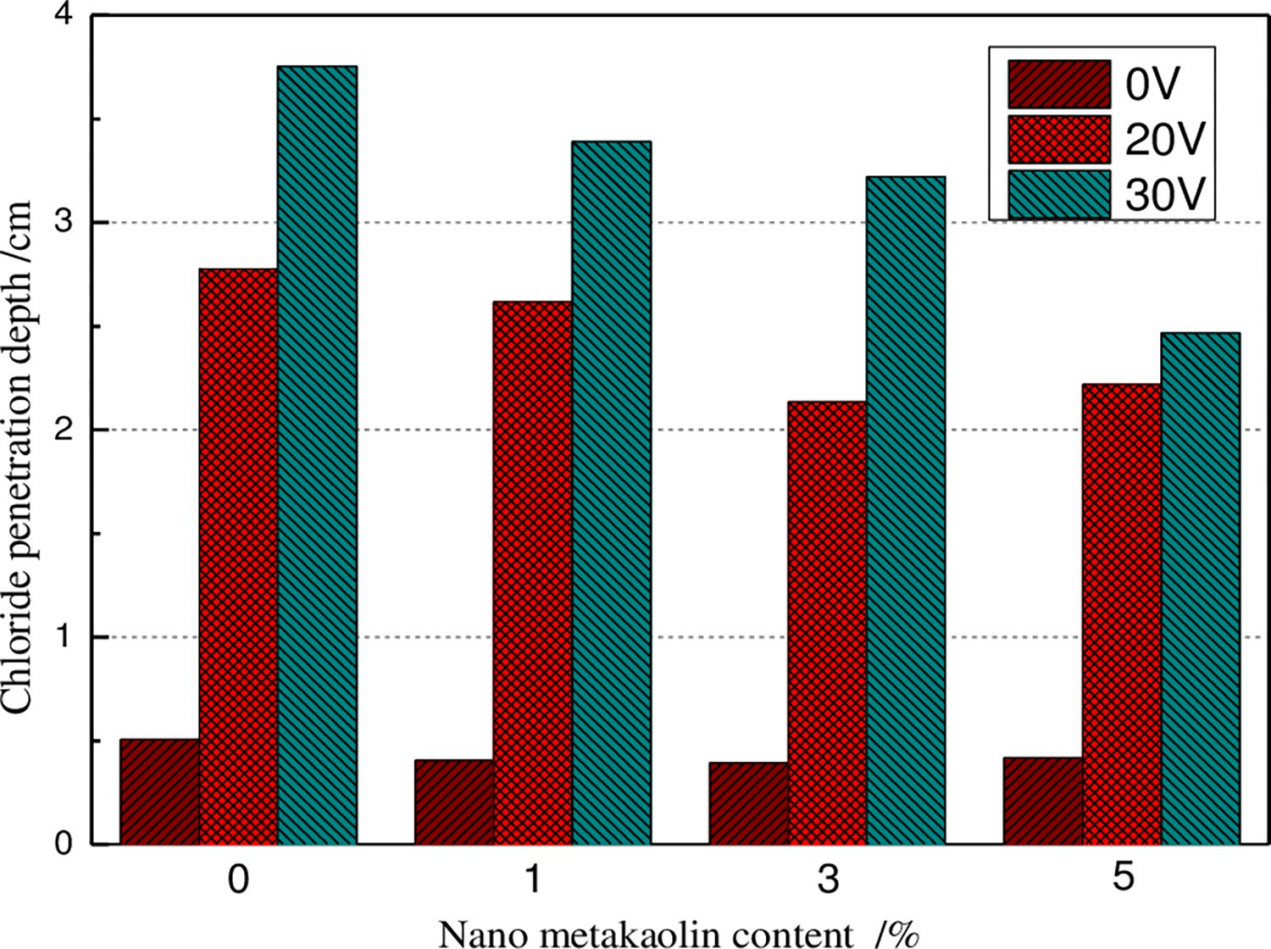
Chloride diffusion depth of GO added concrete under the electric current.

The change of chloride ion penetration depth of GO concrete in the presence of various applied voltage accelerated corrosion has been illustrated in Fig 5 for 36 hours.

Under natural infiltration conditions (i.e., the accelerated corrosion voltage is 0 V, as presented in Fig 5), the chloride ion penetration depth of concrete lessens first and then growth with the increase of GO content. The chloride ion penetration depth of 3% GO concrete is the smallest, which is 22.38% less than that of ordinary concrete specimens. The chloride penetration depth of concrete with 5% GO is 17.43% less than that of ordinary concrete specimens. When the accelerated corrosion voltage is set equal to 20 V, the variation law of chloride ion penetration depth of concrete becomes similar to that of natural penetration. Additionally, the chloride ion penetration depth of 3% and 5% GO concrete is 23.13% and 20.10% less than that of ordinary concrete, respectively. When the corrosion acceleration voltage is set equal to 30 V, concrete specimens’ chloride ion penetration depth decreases with the increase of the GO content. The chloride ion penetration depth of 5% GO concrete is about 34.25% less than that of ordinary concrete specimens. Therefore, the addition of GO could effectively reduce both the migration process and penetration of chloride ions in concrete. The reason is mainly due to the filling effect of the GO on the concrete matrix and the promotion of hydration, which lessens the internal porosity of concrete, reduces the ion concentration of pore solution, and slows down the migration process of chloride ions, and reduces the penetration depth of chloride ions. The demonstrated results in Fig 5 indicate that the chloride ion penetration depth of GO concrete increases with the grows of applied voltage, indicating that the greater the applied voltage, the stronger the ion current inside the concrete, and the faster the chloride ion penetration rate inside the concrete, and then the chloride ion penetration depth increases.

**Fig 6:**
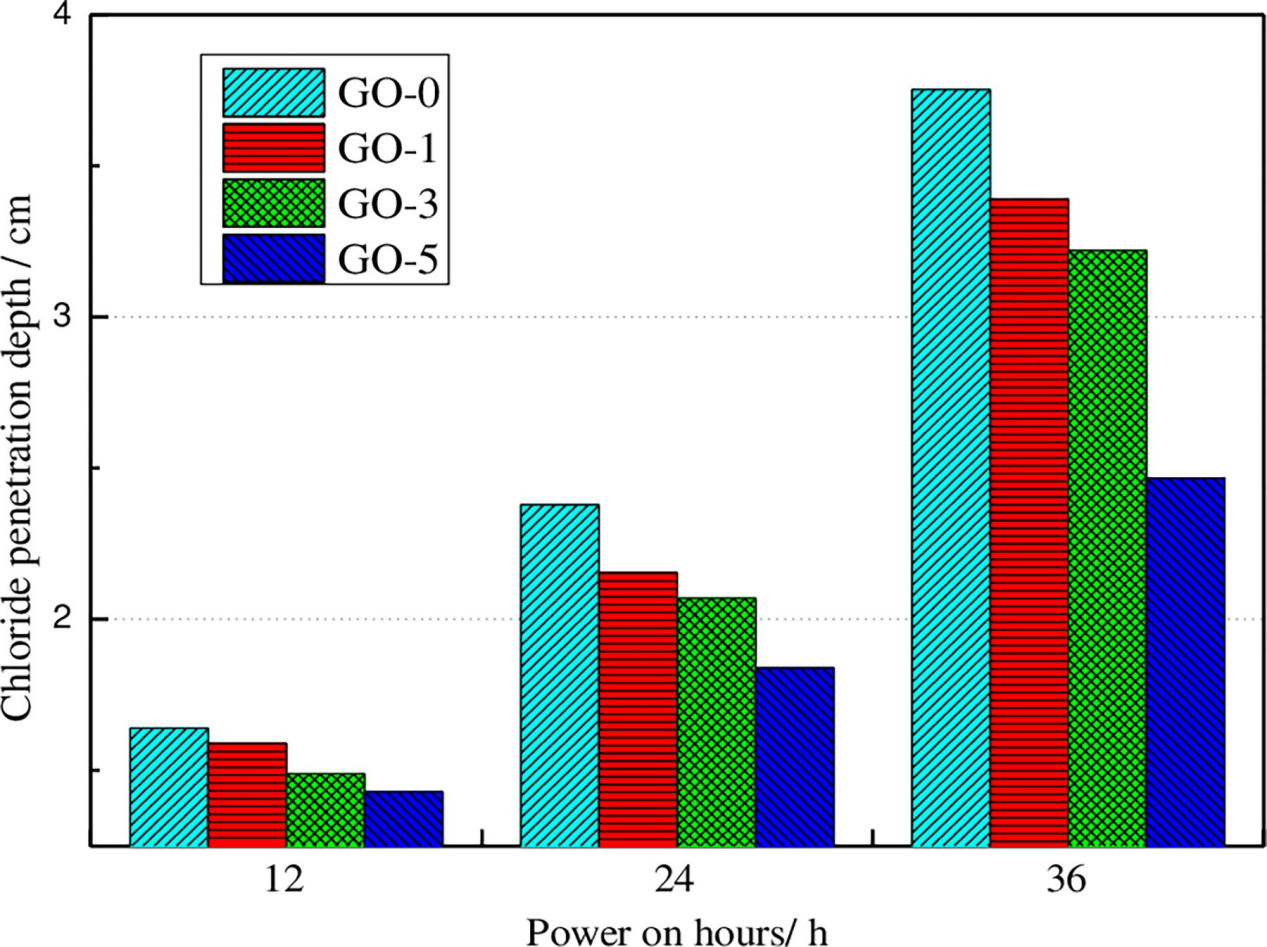
Chloride diffusion depths of GO added concrete under the various applied current period.

Under the accelerated corrosion voltage of 30 V, the variation of chloride ion penetration depth of GO concrete after different corrosion times has been presented in Fig 6. Under natural infiltration conditions, concrete’s chloride ion penetration depth decreases first and then increases as the increase of GO content. The chloride ion penetration depth of 3% GO concrete exhibits the smallest level, which is 22.38% lower than that of ordinary concrete specimens. The chloride penetration depth of concrete with 5% GO is 17.43% lower than that of ordinary concrete specimens. When the accelerated corrosion voltage is set equal to 20 V, the variation law of the chloride ion penetration depth of concrete is similar to that of the natural penetration. The chloride ion penetration depth of 3% and 5% GO concrete in order are 23.13% and 20.10% lower than that of ordinary concrete. When the corrosion acceleration voltage is set as 30 V, the chloride ion penetration depth of concrete specimens lessens with the increase of GO content. The chloride ion penetration depth of 5% GO concrete is 34.25% lower than that of ordinary concrete specimens. Therefore, GO could effectively reduce the migration process of chloride ions in concrete and the penetration of chloride ions in concrete. The reason is mainly due to the filling effect of GO on the concrete matrix and the promotion of hydration, which reduces the internal porosity of concrete, reduces the ion concentration of pore solution, retards the migration process of chloride ions, and reduces their penetration depth of chloride ions. The plotted results in Fig 5 indicate that the chloride ion penetration depth of GO concrete growth with the increase of the applied voltage, indicating that the greater the applied voltage, the stronger the ion current inside the concrete, and the faster the chloride ion penetration rate inside the concrete, and then the chloride ion penetration depth increases.

Under the action of accelerated corrosion voltage of 30 V, the variation of chloride ion penetration depth of GO concrete after various corrosion times has been demonstrated in Fig 6.

Fig 6 displays that under different accelerated corrosion times, chloride ion penetration depth of concrete decreases with the increase of GO content. The chloride ion penetration depth of 1%, 3%, and 5% GO concrete specimens lessens by 9.65%, 14.15%, and 34.25%, respectively, compared with ordinary concrete specimens after accelerated corrosion for 36 hours. The chloride ion penetration depth of GO concrete specimens increases with the increase of accelerated corrosion time, which is due to the increase of chloride ion migration in concrete with the extension of accelerated corrosion time, which increases the chloride ion penetration depth.

#### 3.2.2. Chloride ion content in concrete

**Fig 7:**
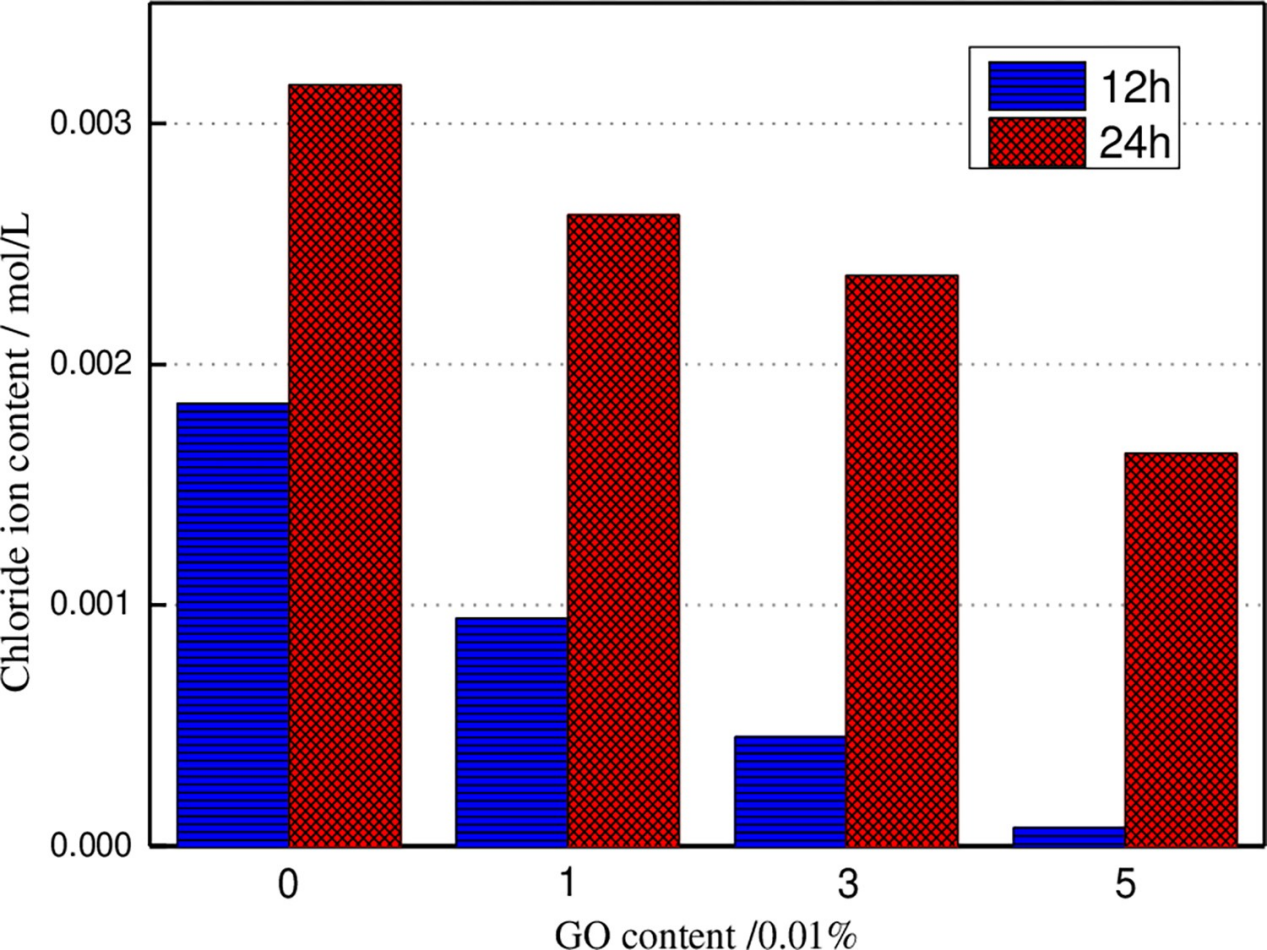
The chloride content in concrete specimens under the various applied current periods.

After 30V accelerated voltage corrosion, the chloride ion content in concrete with 5mm penetration depth changes, as illustrated in Fig 7. It can be seen that the longer the accelerated corrosion time, the more the chloride ion content in concrete, and the chloride ion content in concrete decreases with the growth of the GO content. After 24 h of accelerated corrosion, the internal chloride ion content of concrete specimens with 5% GO is 48.42% lower than that of ordinary concrete. The incorporation of GO reduces the internal pore structure of the concrete matrix, slows down the chloride ion intrusion process, and reduces the chloride ion content in the pore solution of the concrete matrix.

**Fig 8:**
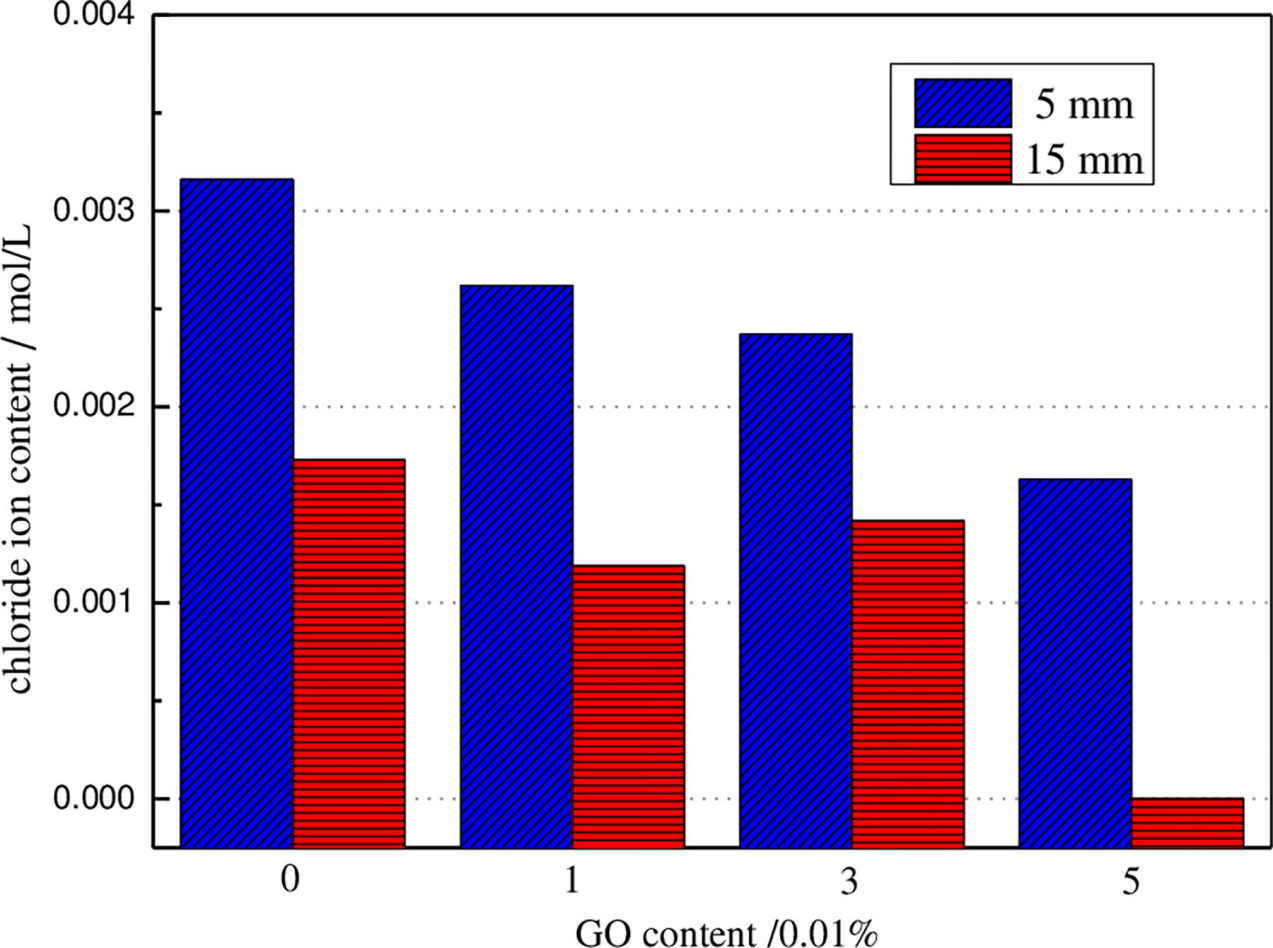
The relationships between the amount of GO and chloride content in concrete.

After 30V accelerated voltage corrosion for 24 hours, the chloride ion contents in concrete with a penetration depth of 5mm and 15mm are presented in Fig 8. The plotted results show that the larger the distance from the concrete specimen surface, the smaller the chloride ion content in the concrete. With the gradual accumulation of chloride ions on the surface of the concrete, the chloride ion concentration on the outer surface of the concrete gradually increases, and the driving force caused by the difference in chloride ion concentration gradually increases, which promotes the gradual migration of chloride ions to the inside. Therefore, the greater the distance between the inside of the concrete and its surface, the lower the amount of chloride ion.

### 3.3. GO concrete underthe action of freeze-thaw

#### 3.3.1. Appearance deterioration characteristics

**Fig 9:**
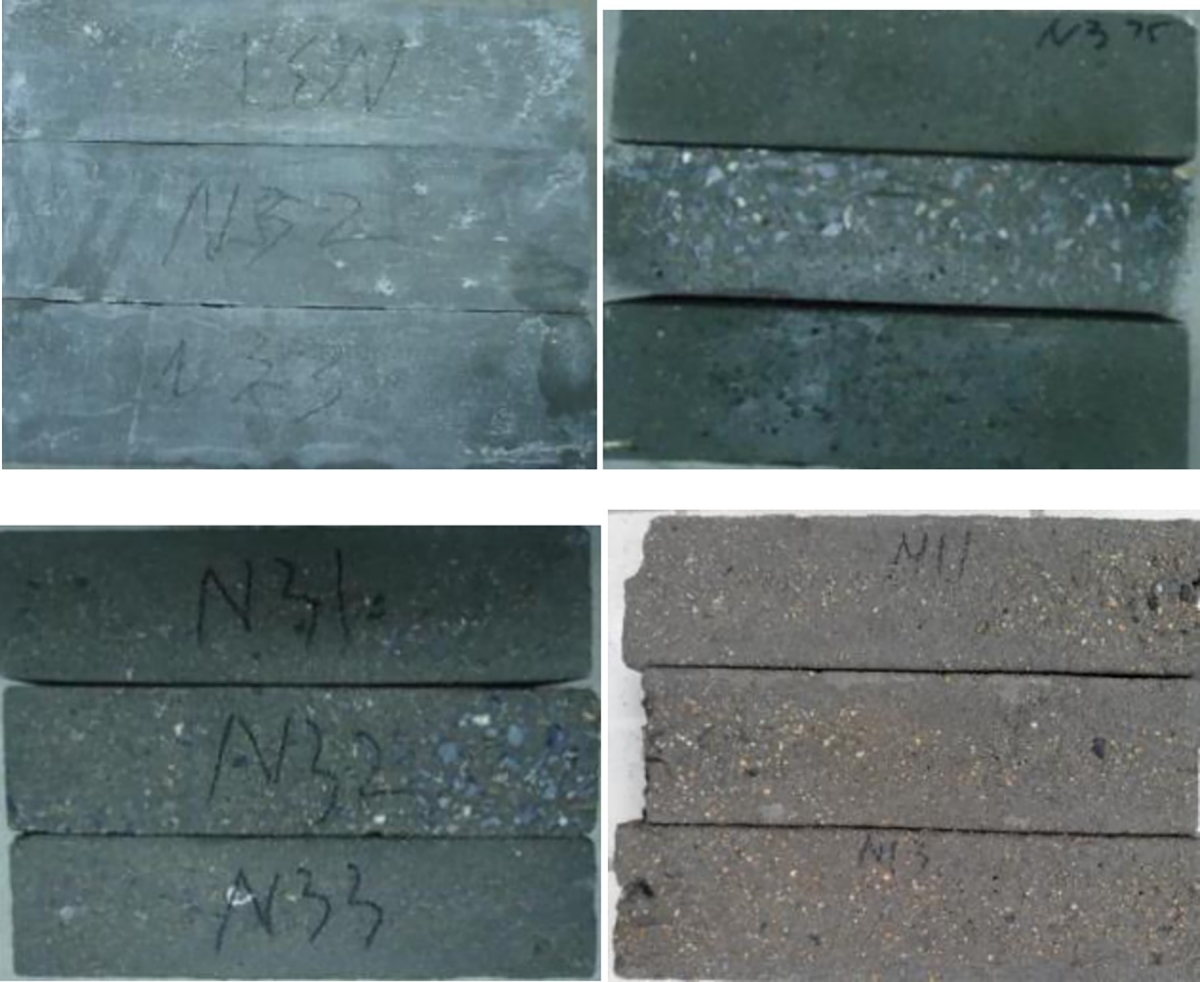
Surface description of plain concrete exposed to different freeze-thaw cycles.

**Fig 10:**
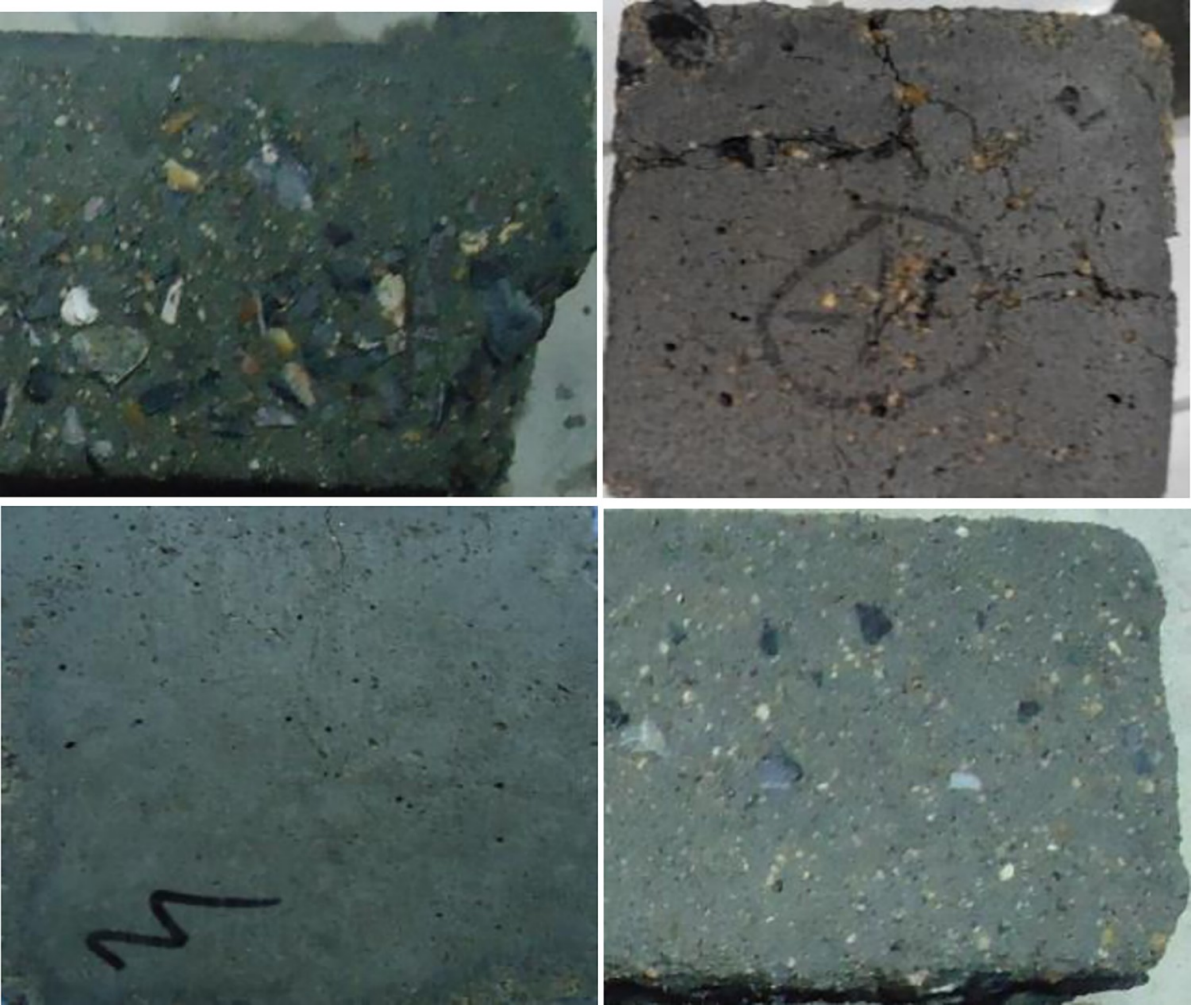
Concrete specimens with different GO exposed to the freeze-thaw cycles 200 times.

The appearance change characteristics of GO concrete specimens under various freeze-thaw cycles are demonstrated in Figs 9 and 10. The graphed results indicate that the GO concrete is corroded under freeze-thaw action. At the initial stage of freeze-thaw, the surface of concrete specimens fell off from a small amount of micro-particle cement slurry until the surface of floating slurry fell off in large areas, where the particle sizes were in the range of 2 ~ 3mm.With the increase of freeze-thaw cycles, when the number of cyclesreaches100, the amount of slurry erosion on the surface of the specimen further intensifies, the fine aggregate is gradually exposed, and the coarse aggregate is bulged. The micropores at the corner gradually become larger and larger, and the local corner is flaked off. Simultaneously, the macroscopic microcracks appear at the interface of the surface aggregate of the specimen. When the freeze-thaw cycle reaches 150 times, the fine aggregate of the surface of the specimen is layered off, and the particle size is developed to several centimeters. The corners and surfaces become uneven, and the number of pores grows. The internal microcracks continue to expand. After 200 freeze-thaw cycles, the phenomenon of falling slag at the corner of the specimen occurs, the coarse aggregate is exposed obviously, and the cohesive force at the aggregate interface decreases. The structure becomes crisp and loose, and the freeze-thaw damage to the specimen is severe.

The presented results in Fig 10 reveal that when the number of freeze-thaw cycles reached200, the surface cementitious materials of GO-0, GO-1, and GO-5 specimens of GO concrete were hugely lost, with more exposed gravels, more and more bottomless freezing pits on the surface, and the blocking phenomenon appeared at the corner around the specimen. There was only a small amount of exposed gravel on the surface of GO-3 specimen, with fewer freezing pits on the surface, and there was no peeling phenomenon at the aggregate boundary. The denudation of the GO-5 specimen was more severe than that of GO-3.

#### 3.3.2. Mass loss

Mass loss is a crucial index of concrete damage during freeze-thaw cycles. Based on the visual observations described above, it was apparent that honeycomb cavities were formed as freeze-thaw cycles continued, causing the mass to change. The mass values for all of the specimens during the freeze-thaw cycles were obtained. The damage sustained by the concrete specimens subjected to the freeze-thaw cycles was evaluated using a mass loss ratio, which is defined as,

$$D_{c}=(1-\frac{m_{cn}}{m_{c0}})\times 100\%$$ (3)

where *D*_*c*_ is the mass loss ratio of the specimen after suffering freeze-thaw cycles; *m*_*cn*_ is the mass of the specimen after exposure to the *N*th freeze-thaw cycles; and *m*_*c0*_ is the mass of the specimen before the freeze-thaw test.

**Fig 11:**
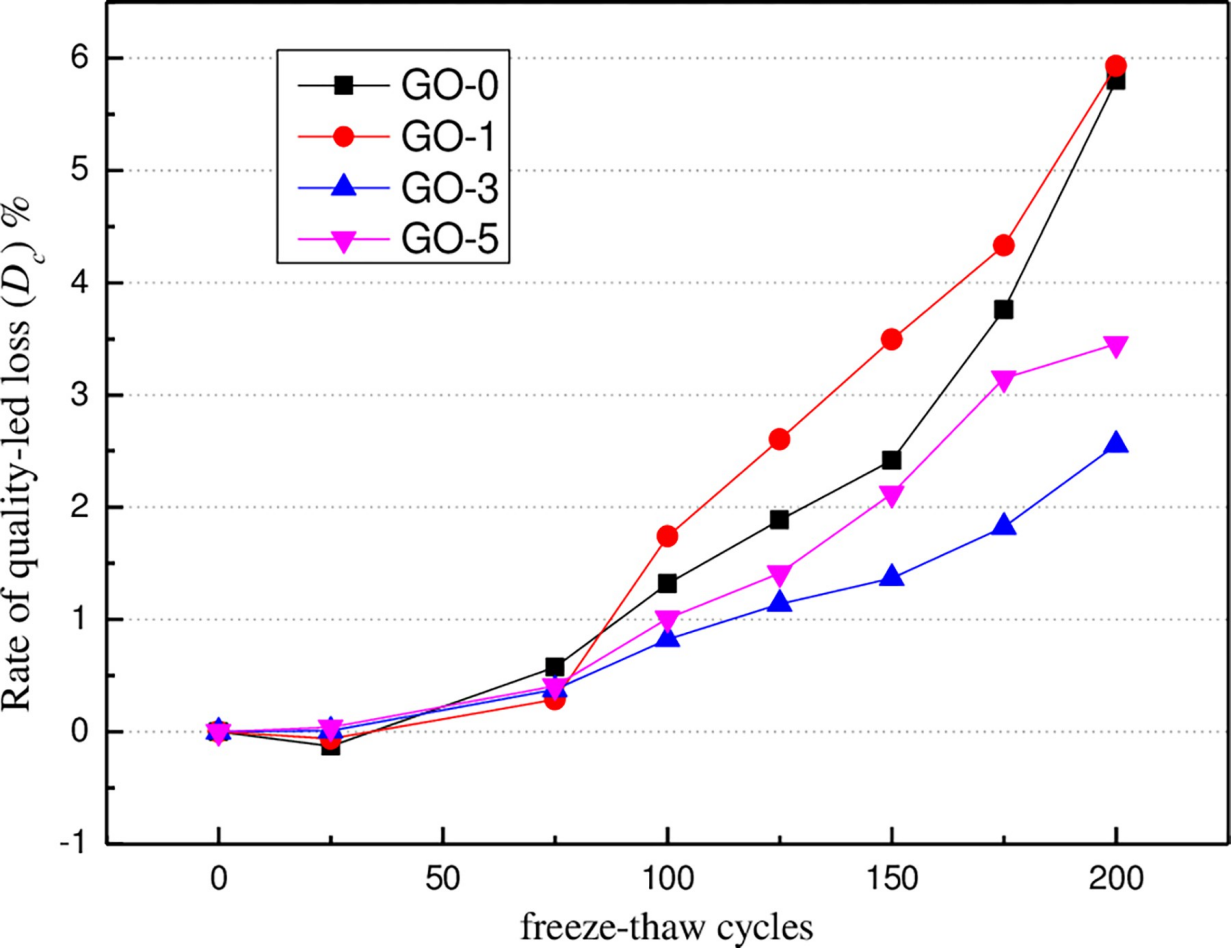
Changes in mass loss of GO-modified concrete under different freeze-thaw cycles.

The variation law of mass loss of concrete specimens during freeze-thaw cycles is illustrated in Fig 11. The mass loss rate of specimens is the average value of the mass loss rate of three specimens in this group. It can be seen from the figure that the mass loss of specimens reduces with the growth of the number of freeze-thaw cycles from 0 to 25 in the initial stage of freeze-thaw. After 25 freeze-thaw cycles, the mass loss rate of specimens gradually increases with the increase of freeze-thaw cycles. The main reasons for this issue are given as follows. At the beginning of the freeze-thaw cycle, microcracks appear in the specimen due to the effect of pore water inside the specimen, and crack development takes place quickly. As a result, the newly added water absorption quality inside the specimen is more excellent than that of the concrete surface. Until 25 freeze-thaw cycles, the loss quality of the cement paste reaches the balance with the water absorption quality, and the mass of the specimen reaches the maximum. With the growth of freeze-thaw cycles, the surface and corner of the specimen become crisp, resulting in surface erosion and even block phenomenon, which leads to a reduction in specimen quality, particularly after more than 100 freeze-thaw cycles, this phenomenon is more serious. Compared with GO-0, the mass loss rates of GO-1 and GO-5 specimens are noticeably increased, while the smallest surface shedding is detected forGO-3 (0.03%) concrete specimens, which can effectively improve the frost resistance of concrete.

#### 3.3.3. Mechanical property

**Fig 12:**
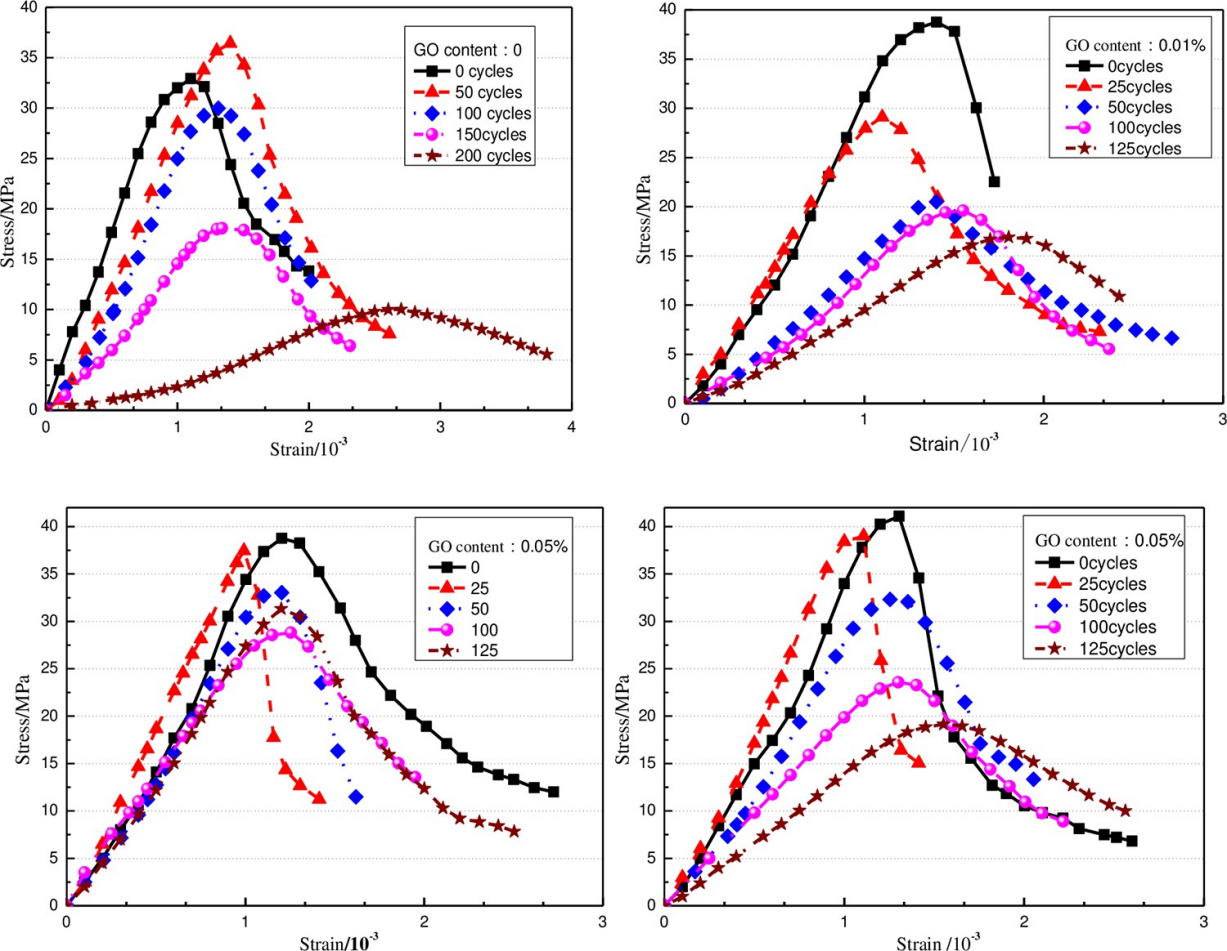
Stress-strain relationship of concrete under freeze-thaw cycles.

The stress-strain curves of GO concrete under various freeze-thaw cycles are obtained, as demonstrated in Fig 12. The plotted results show the increase in freeze-thaw times. The peak point decreases to move right, and the stress-strain curve tends to be flat. This fact is mainly due to the freeze-thaw action, the inside and surface of concrete specimens have different degrees of damage, and the macro performance is that the peak strain gradually increases and the peak stress gradually decreases. The elastic modulus gradually decreases. The stress-strain curves of different GO concrete specimens are slightly different. Compared with ordinary concrete, the flat speed of the curve of 0.03% GO concrete specimen is relatively slow, mainly because the addition of GO changes the concrete microstructure, resulting in weak damage and failure of the specimen during freezing and thawing.

### 3.4. Study on modification mechanism of GO

#### 3.4.1Microstructure analysis

**Fig 13:**
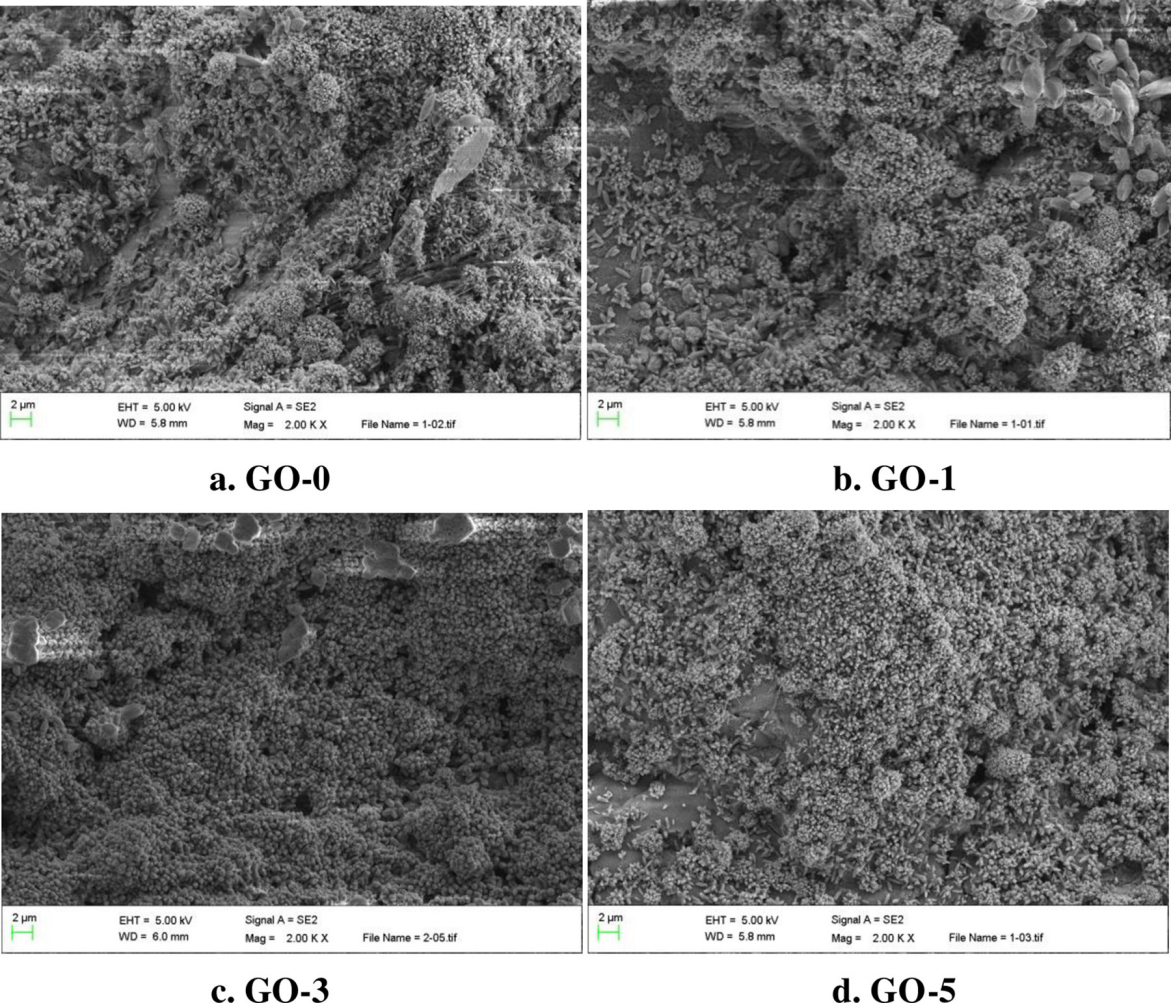
Microstructure of specimens after 28 days curing.

Fig 13 presents the microstructure of GO-hardened cement for the curing age of28 days.

It can be seen from Fig 13(A) that the microstructure of cement paste without GO is loose, uneven, and not dense, and there are holes, cracks, and other defects within the specimen. There are few hydration crystal products, needle-like, and lamellar crystal products. The aggregation morphology is irregular, and the distribution is uneven. Fig 13(B) demonstrates that the hydration products are massive accumulation, the crystal is coarse and polyhedral, and the structure is relatively dense; Fig 13(C) shows the microstructure of cement paste based on 0.03% GO. The hydration products of cement paste are extruded into laminated layers, and a small amount of rod-like CH crystals are inserted into the C-S-H gel to make the microstructure denser. With the growth of GO content, Fig 13(D) illustrates the variations of the microstructure of cement paste, a large number of rod-like crystals interweave with each other, and many defects such as pores and cracks appear in the internal structure, which reduces the mechanical properties of cement paste. Therefore, with the addition of a small amount of GO, the crystal products in cement paste are considerably grow, and the microstructure becomes compact, but there are still a few defects, such as pores and cracks.

#### 3.4.2XRD analysis

**Fig 14:**
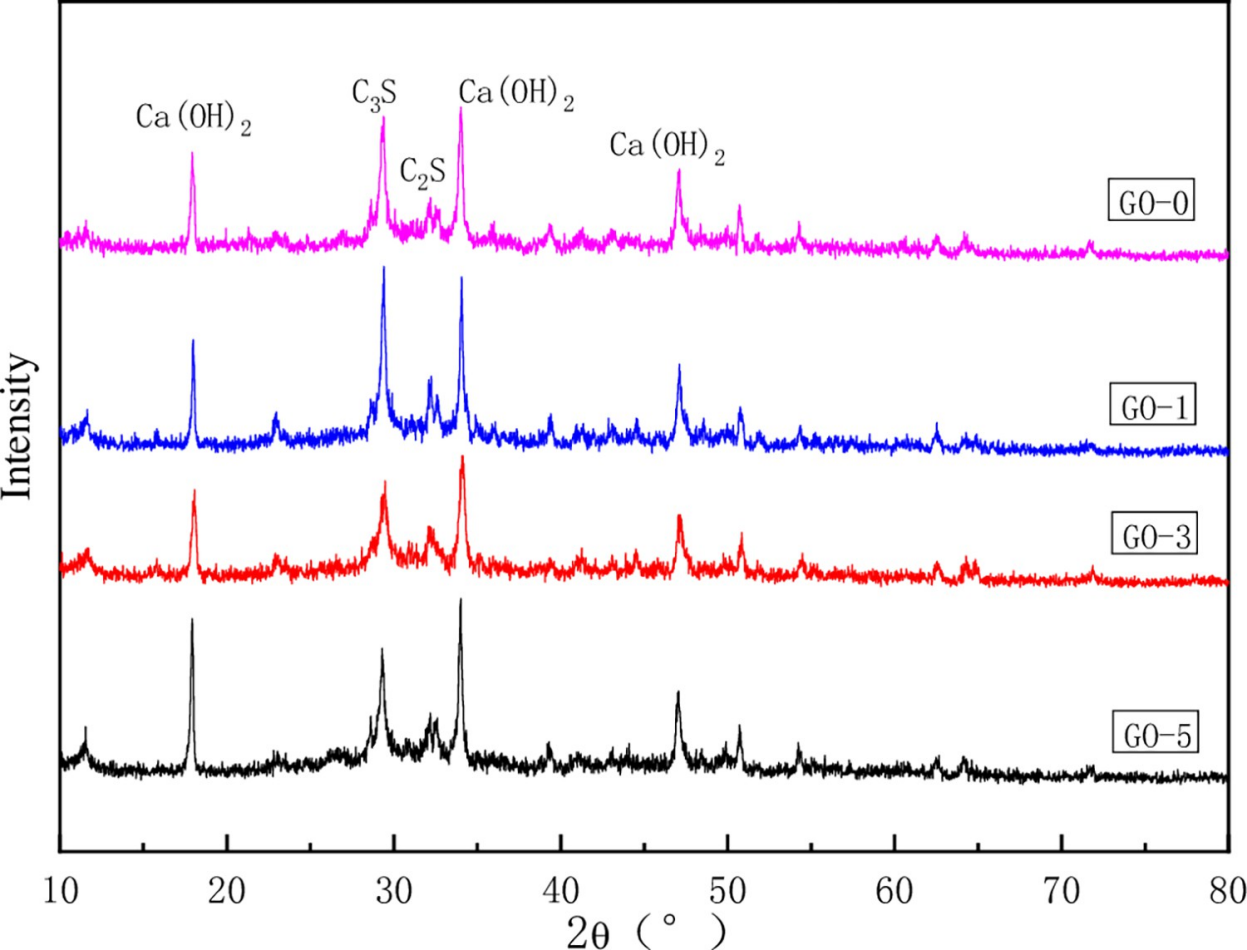
XRD of specimens after 28 days curing.

In order to explore the effect of GO on the hydration products of cement paste, the types and changes of crystal phases in hydration products are characterized by XRD (see Fig 14).

The demonstrated plots in Fig 14 indicate that the crystal structures of GO cement paste with various contents are the same as those of ordinary cement pastes. The main components of hydration products are CH crystal, Aft crystal, AFM crystal, and C-S-H. The difference is that the crystal absorption peak strength of ordinary cement pastes, the gel composition, and the structure are orderly. Therefore, the addition of GO altered the microstructure of hydration products and played an assembly and template role in the hydration process of cement paste.

From the above analysis and discussion, it can be seen that the microstructure of cement hydration products under conventional conditions is wholly different from that of cement hydration crystal products with GO. The incorporation of GO plays a promoting role in the formation and shape of cement hydration crystal products. First, the oxygen-containing groups on the surface of GO reacted with the active components C_2_S, C_3_S, C_3_A, and C_4_AF in cement to form the growth point of hydration crystals. With the continuous hydration reaction, the columnar crystals were configured on the surface of GO sheets. Under the control of GO assembly, multiple columnar crystals wrapped each other to configure coarse crystal products, which reduced the pores and cracks in the hydration crystal products, and formed a polyhedral hydration crystal aggregation with mutual superposition and interweave. However, the hydration products of ordinary cement paste were chaotic, and there were many pores and cracks in the microstructure. Therefore, GO has the function of assembling and regulating the shape of hydration crystals, which could intertwine and bind the hydration crystal products to form polyhedral hydration products, and the microstructure is more compact, regular, and orderly.

#### 3.4.3 DSC analysis

**Fig 15:**
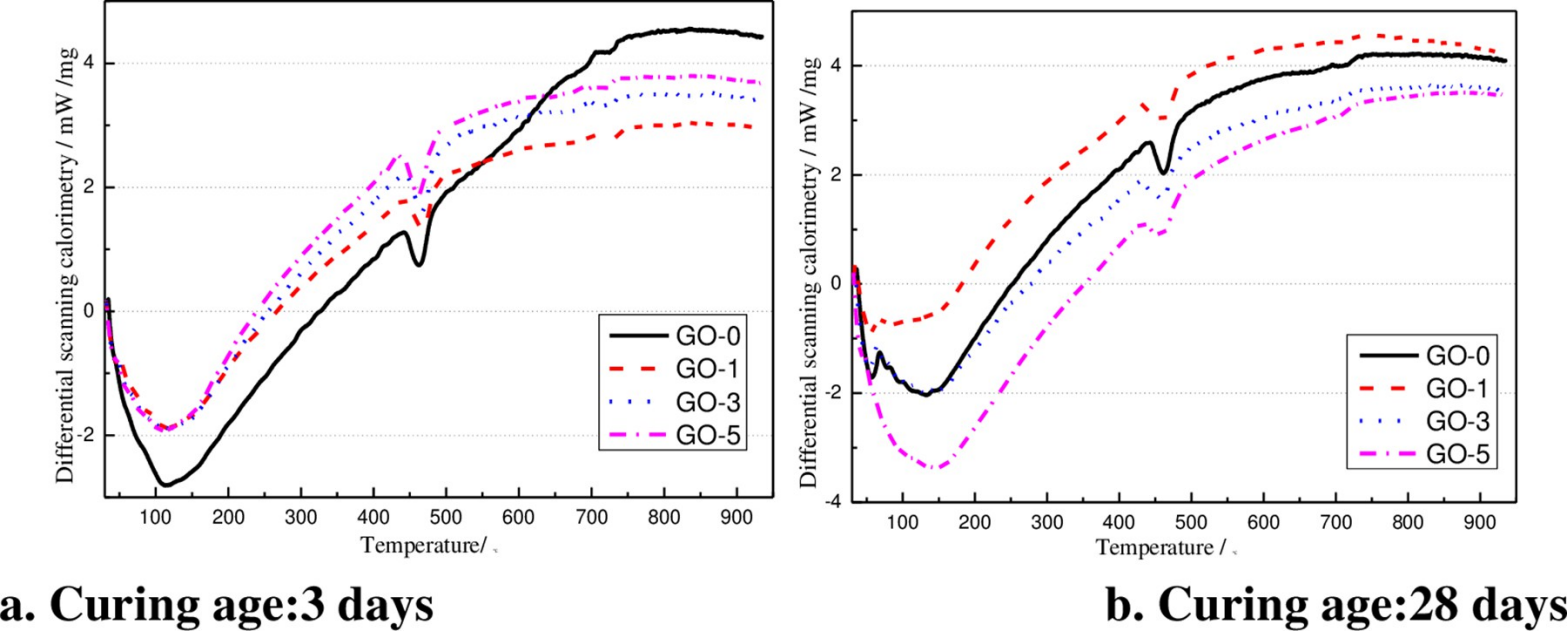
DSC thermograms of the cement pastes with the addition of GO at various curing ages.

After different curing ages (i.e.,3dand 28 d), the DSC curves of GO cement slurry GO-0, GO-1, GO-3, and GO-5 are provided in Fig 15.The presented graphs in Fig 15 reveal that after 3 days of hydration reaction, noticeable weight loss phenomenon occurred in the temperature ranges of 50°C ~ 150°C, 400°C ~ 480°C, and 650°C ~ 750°C. It implies that after 3 days of hydration reaction, more hydration products C-S-H gelling, and CH crystals were produced. The weight loss of ordinary cement slurry was more obvious than cement slurry doped with GO-3 and GO-5, and the degree of hydration reaction was GO-1 > GO-0 > GO-5 > GO-3. During curing up to 28 d, the weight loss curve of the GO-doped cement paste was not significantly different from that of ordinary concrete, the hydration reaction process slowed down, and the hydration tended to complete.

**Fig 16:**
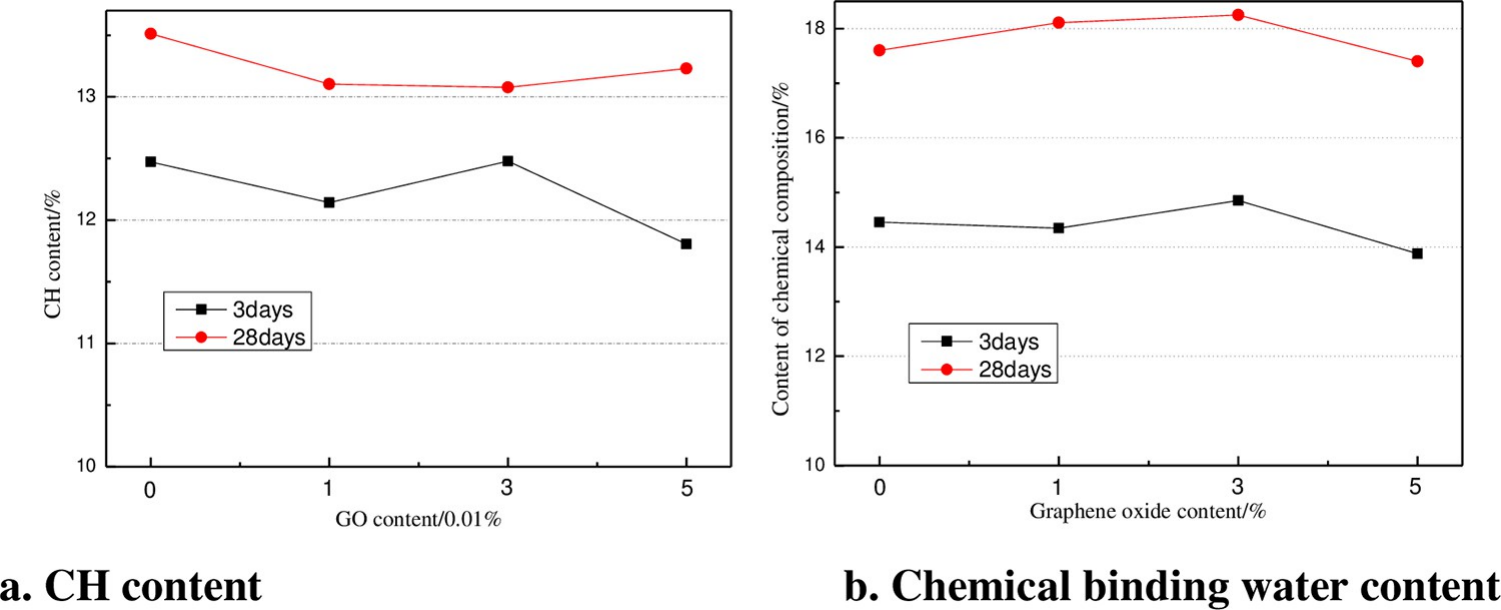
Contents of CH and chemical binding water of GO cement slurry at various curing ages.

The contents of CH and chemical binding water in GO-0, GO-1, GO-3, and GO-5 cement slurry are provided in Fig 16. The given results in Fig 16 indicate that at the age of 3d, the CH crystals in the cement paste with 0.01% and 0.05% GO were less than those in the ordinary cement paste. The addition of 0.03% GO leads to the increase of the CH crystals in the cement paste, and the CH crystals were enhanced by 1.2%. After curing for 28 d, the CH crystal of cement slurry doped with GO lessened compared with that of ordinary cement slurry, and the CH crystal doped with 0.05% GO lessened by 7.2% compared with that of ordinary cement slurry. The chemical water binding capacity of cement slurry with 0.01% and 0.03% GO was enhanced, and the chemical water binding capacity of cement slurry with 0.05% GO was less than that of ordinary cement slurry. It reveals that the GO could effectively promote the hydration process of cement, as well as the formation of C-S-H gel by CH crystal. In the case of the addition of 0.03% GO, the template effect of GO could be fully exerted, and the microstructure and basic properties of cement-based materials could be enhanced.

## 4 Conclusions

1. The addition of an appropriate amount of GO could effectively enhance the early mechanical properties of the cement paste. The 7d flexural strength of the cement paste with 0.03% GO was 22.71% higher than that of the ordinary cement paste, and the compressive strength was increased by 22.71%.
2. Under accelerated corrosion conditions, the penetration depth of chloride ions and the ircontent in the same part lessened with the growth of GO content.
3. After freezing and thawing, the compressive strength of GO concrete specimens with different contents was enhanced with the growth of GO contents. The compressive strength was increased by 29.3%. Therefore, incorporating GO could improve the compressive strength of salt-frozen concrete. The stress-strain curves of different GO concrete specimens were slightly different. Compared with ordinary concrete mixed with 0.03% GO, the flattening speed of the curve of the concrete specimen was relatively slow. Therefore, the addition of GO could enhance the ability of concrete to resist freeze-thaw deformation.
4. Microscopic explorations reveal that the GO can promote cement hydration and has the effect of assembling and regulating cement hydration crystals. On the GO sheet, the hydration crystal products are intertwined to form polyhedral hydration products, and the microstructure is regular and orderly denser. Adding a small amount of GO can promote the cement hydration process, and the chemical water binding amounts in the cement slurry with 0.01% and 0.03% GO in order were 2.16% and 2.22% higher than those in the ordinary cement slurry.

## Supporting information

S1 Data (XLSX)
